# Avian Leukosis Virus Subgroup J Attenuates Type I Interferon Production Through Blocking IκB Phosphorylation

**DOI:** 10.3389/fmicb.2018.01089

**Published:** 2018-05-25

**Authors:** Wencheng Lin, Zhouyi Xu, Yiming Yan, Huanmin Zhang, Hongxin Li, Weiguo Chen, Feng Chen, Qingmei Xie

**Affiliations:** ^1^Guangdong Provincial Key Lab of Agro-Animal Genomics and Molecular Breeding and Key Laboratory of Chicken Genetics, Breeding and Reproduction, Ministry of Agriculture, College of Animal Science, South China Agricultural University, Guangzhou, China; ^2^South China Collaborative Innovation Center for Poultry Disease Control and Product Safety, Guangzhou, China; ^3^Key Laboratory of Animal Health Aquaculture and Environmental Control, Guangzhou, China; ^4^Avian Disease and Oncology Laboratory, USDA, Agriculture Research Service, East Lansing, MI, United States

**Keywords:** ALV-J, immunosuppression, macrophage, type I interferon, NF-κB

## Abstract

Avian leukosis virus subgroup J (ALV-J) is an oncogenic retrovirus that causes immunosuppression and enhances susceptibility to secondary infection, resulting in great economic losses. Although ALV-J-induced immunosuppression has been well established, the underlying molecular mechanism for such induction is still unclear. Here, we report that the inhibitory effect of ALV-J infection on type I interferon expression is associated with the down-regulation of transcriptional regulator NF-κB in host cells. We found that ALV-J possess the inhibitory effect on type I interferon production in HD11 cells and that ALV-J causes the up-regulation of IκBα and down-regulation of NF-κB p65, and that ALV-J blocks the phosphorylation of IκBα on Ser32/36 amino acid residues. Collectively, our findings provide insights into the pathogenesis of ALV-J.

## Introduction

Avian leukosis virus subgroup J (ALV-J) is a prototypical member of the genus *Alpharetrovirus* of the sub-family *Orthoretrovirinae* of family *Retroviridae*, causing diverse tumors, such as myelocytomas, myeloid leukosis, sarcomas, hemangiomas, nephromas and erythroblastosis, and resulting in great economic losses (Stedman and Brown, 1999; Payne and Nair, 2012). In addition, ALV-J also causes immunosuppression and enhances susceptibility to secondary infection (Justice et al., 2015). Although ALV-J-induced immunosuppression has been well established, our understanding of the exact molecular mechanisms of ALV-J-induced immunosuppression is still limited.

The innate immune system is considered as the first line of host defense against viral infection, causing signal transduction to induce protective cellular genes (Chen et al., 2003). Rapid induction of type I interferon (IFN) and inflammatory cytokines are central to the host antiviral responses, which are tightly regulated by extracellular and intracellular signals (Huang et al., 2003). Type I interferon including IFN-α and IFN-β play a critical role in antiviral innate immunity and in modulating the adaptive immune response to viral infection, which is usually regulated by Nuclear Factor-κB (NF-κB) signaling pathway (Wang et al., 2010).

NF-κB is a homo- or hetero-dimeric complex composed of members of the Rel family of DNA-binding proteins, inducing the activation of a large number of genes in response to pathogen infections, immunity, inflammation, cell growth, tumorigenesis and apoptosis. NF-κB is sequestered in the cytoplasm as an inactive form via interaction with the IκB family of inhibitory proteins in the canonical NF-κB signaling pathway (Baldwin, 1996; Napetschnig and Wu, 2013). In response to extracellular stimuli including cytokines, viruses, bacteria, oxidative stress, and DNA damaging agents, the interaction between NF-κB and the IκB family of inhibitory proteins is usually dissociated following the phosphorylation of the IκBs, and the free NF-κB dimers translocate into the nucleus to activate the transcription of a subset of genes encoding inflammatory and immunoregulatory molecules (Nomura et al., 2000; Hacker and Karin, 2006; Kawai and Akira, 2007).

It has been reported that ALV-J-induced immunosuppression is involved in multiple aspects of the pathogenesis, such as cytokine regulation, the pro-inflammatory response, apoptosis and adaptive immune response (Feng and Zhang, 2016; Feng et al., 2016; Liu et al., 2016b; Feng M. et al., 2017). In the present study, we investigated whether and how ALV-J overcomes host antiviral innate immunity by mRNA sequencing (mRNA-Seq), luciferase reporter gene assay and quantitative real-time PCR (qRT-PCR). We found that ALV-J possesses the inhibitory effect on type I interferon production in host cells, and the inhibitory effect might be associated with the activation of NF-κB signaling pathway. We also observed that ALV-J inhibits the expression of NF-κB and blocks IκBα phosphorylation on Ser32/36 amino acid residues, which might stabilize the NF-κB/IκB complex and prevent the transfer of NF-κB into the nucleus. Our study provides a basis for research into the mechanisms behind the pathogenicity of ALV-J.

## Materials and Methods

### Cell Lines and Virus

HD11 cells (an chicken macrophage-like cell line) kindly provided by Dr. Liu Jue (Beijing Academy of Agriculture and Forestry, Beijing, China) were cultured in RPMI 1640 supplemented with 10% fetal bovine serum (FBS). DF-1 (immortal chicken embryo fibroblast) cells were cultured in Dulbecco’s Modified Eagle Medium (DMEM) supplemented with 10% FBS. The ALV-J NX0101 strain used in this study was kindly provided by Dr. Cui Zhizhong (Shandong Agricultural University).

### Quantification of ALV-J Replication in HD11 Cells

HD11 cells were seeded in cell culture plates at 3 × 10^5^ cells/mL. When cells had grown into a monolayer, they were infected with a dose of 10^3^TCID_50_ of ALV-J NX0101 strain. Cells were incubated with RPMI 1640 under the same incubation condition as a negative control. At 0, 6, 12, 24, 36, and 48 h post infection (hpi), viral genomic RNA was extracted from mock- and ALV-J-infected cells using TRIzol reagent (Invitrogen Corp.) according to the manufacturer’s instruction. The qRT-PCR was performed using the specific primer pairs for ALV-J gp85 as described previously (Dai et al., 2015). The analysis of real-time PCR was carried out with a CFX96 Touch (Bio-Rad).

### TCID_50_ Assay

HD11 cells were infected with a dose of 10^3^TCID_50_ of ALV-J NX0101 strain or medium as a control, and cell cultures were collected at 0, 6, 12, 24, 36, and 48 hpi. Virus were titrated using 50% tissue culture infective doses (TCID_50_) as described previously (Qin et al., 2013). Briefly, viral solution harvested at different time points were serially diluted 10-fold in RPMI 1640. A 100-μL aliquot of each diluted sample was added to the wells of multiple 96-well plates, followed by addition of 100 μL of DF-1 cells at a density of 2.5 × 10^5^ cells/mL. Cells cultured for 48 h at 37°C in 5% CO_2_ were fixed with 4% paraformaldehyde and permeabilized using 0.1% Triton X-100, blocked by bovine serum albumin, and probed with the specific anti-gp85 antibody and an FITC-conjugated goat anti-mouse IgG antibody. The cells were stained with DAPI, and enumerated by fluorescence microscopy. Viral titers were determined according to the Reed and Muench method (Reed, 1938).

### The Effect of ALV-J on Type I Interferon Production in HD11 Cells

HD11 cells or DF-1 cells grown into a monolayer were infected with ALV-J or medium as a control, and then treated with TNF-α or Poly (I:C) or with DMEM medium as a control. Cells were harvested at 6, 12 and 24 hpi. Total RNA were isolated using TRIzol reagent and reverse-transcribed using reverse transcriptase according to the manufacturer’s protocol (GeneCopoeia, MD, United States). The specific primers for chicken IFN-α1, IFN-β, NF-κB p65, and GAPDH were designed according to the previous publication (Li Z. et al., 2013). The qRT-PCR was performed in a 20 μL volume containing 10 μl of 2 × SYBR green Premix *Ex Taq*, 1 μL of cDNA template, and a 0.5 μM concentration of specific primers. Thermal cycling parameters were as follows: 95°C for 5 min; 40 cycles of 95°C for 10 s, 56°C for 30 s, and 72°C for 30 s and 1 cycle of 95°C for 30 s, 60°C for 30 s, and 95°C for 30 s. All the samples were reacted in triplicate on the same plate, and the GAPDH gene was utilized as the reference gene. Expression levels of genes were calculated relative to the expression of the GAPDH gene and expressed as fold increase or decrease relative to the control samples.

### The Effect of Poly (I:C) on ALV-J Replication

DF-1 cells were seeded in cell culture plates at 2.5 × 10^5^ cells/mL. When cells had grown into a monolayer, they were treated with Poly(I:C) or with medium as a control prior to ALV-J infection, or infected with ALV-J prior to Poly(I:C) transfection. At 24, 48, and 72 hpi, cell cultures were collected to determine viral loads using AC-ELISA in anti-p27 antibody-coated plates (IDEXX Corp.).

### Luciferase Reporter Assay

Mock- or ALV-J-infected DF-1 cells were transfected with pGL3-chIFNα1 or pGL3-chIFNβ. To normalize for transfection efficiency, pRL-TK Renilla luciferase reporter plasmid was transfected as a control. Twelve hours later, cells were treated with TNF-α or Poly (I:C) or with DMEM medium as a control. Twelve hours after TNF-α or Poly (I:C) treatment, luciferase reporter gene assays were performed using the dual-luciferase assay system (Promega) according to the previous study (Lin et al., 2013).

### mRNA Sequencing Analysis

HD11 cells grown into a monolayer were infected with ALV-J or medium as a control. Twelve hours later, cells were harvested and total RNA was isolated from the mock- and ALV-J-infected cells. The concentration and integrity of the RNA samples were quantified using a Nano-100 instrument (Axiomtek) and denaturing agarose gel electrophoresis (Agilent), respectively. To prepare for the mRNA-Seq, mRNA enrichment, fragmentation, cDNA construction, terminal repair, adding A at the 3′ end, coupling connection, PCR amplification, quality control of the cDNA library, standardization of the cDNA library, and cluster generation was undertaken. Sequencing was performed on an Illumina Hiseq 2500 platform.

Sequencing results were aligned with the chicken reference genome. Expression profile at the gene level was analyzed, the total number of reads mapped to their unigenes were calculated and normalized. Differentially expressed genes were identified with the R package DEGseq, using a Benjamini *q*-value of 0.05 (cut-off at 5% false discovery rate). Differentially expressed genes were selected using the following criteria: fold change ≥2 or ≤0.5 and *P* < 0.05. The DAVID database was used for KEGG and Go enrichment analysis, with the GOseq package, using a corrected *P*-value of <0.05, and ratio ≥1 as the threshold.

According to the mRNA-Seq analysis, differentially expressed genes were verified using qRT-PCR assay. Briefly, HD11 or DF-1 cells grown into a monolayer were infected with ALV-J or medium as a control. Twelve hours later, cells were harvested and total RNA was isolated. The qRT-PCR were performed using specific primer sets (**Table 1**). According to the value obtained for GAPDH, raw data were normalized, the measurement of differentially expressed genes were expressed as the ratio of these mRNA to GAPDH mRNA expression.

**Table 1:** Primer pairs used for qRT-PCR in this study.

Gene symbol	Primer sequence (5′–3′)	Product (bp)	Accession no.
IκBα	F: GCCGCTTTCCTGAACTTC	121	NM_001001472.2
	R: CCCTGACATCCAGGTCGC		
TNFSF10	F: AAAAGGCTGAGTATGGAC	93	XP_015147112.1
	R: CACTGTTACTGACCGAGA		
PIDD1	F: ACAGCTGCACAGAACTGG	198	XP_004941519.2
	R: GGATCTCAGGGGGGACAG		
RNASEL	F: AAGCATTGCCTCTGAGTT	94	XP_015145762.1
	R: TTTGGAGTTCACATCTGC		
IFN-α1	F: CCAGCACCTCGAGCAAT	133	AB021154
	R: GGCGCTGTAATCGTTGTCT		
IFN-β	F: GCCTCCAGCTCCTTCAGAATACG	224	X92479
	R: CTGGATCTGGTTGAGGAGGCTGT		
p65	F: CCACAACACAATGCGCTCTG	112	D13721
	R: AACTCAGCGGCGTCGATG		
ALV-J gp85	F: TGTGTGCGTGGTTATTATTTC	144	DQ115805
	R: AATGGCGAGGTCGCTGACTGC		
GAPDH	F: TGCCATCACAGCCACACAGAAG	123	AF047874.1
	R: ACTTTCCCCACAGCCTTAGCAG		

### Western Blot Analysis

HD11 cells were infected with ALV-J or medium as a control. At 12 hpi, cell lysates were prepared with lysis buffer (1% Triton-100, 5 mM EDTA, 20 mM Tris–Cl, 137 mM NaCl, 0.02% NaN_3_, and 1% protease inhibitor) at 4°C for 30 min, then centrifuged at 13,000 × *g* for 20 min. The samples were fractionated by SDS–PAGE and transferred onto polyvinylidene difluoride membrane. After blocking with 5% skimmed milk, the membranes were incubated with indicated antibodies. Blots were developed using an ECL kit.

### Examination of IκBα Phosphorylation

HD11 cells were infected with ALV-J or medium as a control for the indicated periods. Cell lysates were prepared and examined by Western blot using anti-pIκBα (Ser32/36), anti-IκBα and anti-actin antibodies as previously described (Liu et al., 2015).

### Statistical Analysis

Statistical analyses were performed with the GraphPad Prism (version 5.0) and expressed as means and standard deviation. The statistical significance of data was calculated with one-way ANOVA analysis of variance between the experimental groups. Differences between groups were considered significant at *P* < 0.05.

## Results

### ALV-J Replication in HD11 Cells

**FIGURE 1:**
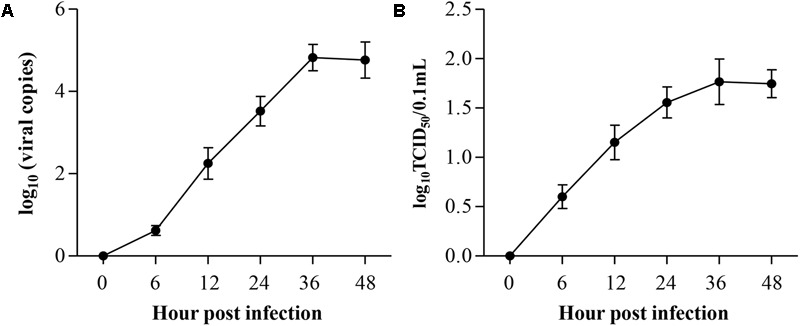
Replication kinetics of ALV-J in HD11 cells. HD11 cells were infected with the indicated dose. At different time points after ALV-J infection, the viral loads were determined by qRT-PCR **(A)** and TCID_50_ assay **(B)**. The graphs show the averages of viral loads from three individual experiments. Results are representative of three independent experiments. Data are represented as mean ± SD, *n* = 3.

Because chicken macrophages are susceptible to ALV-J and because HD11 is a macrophage-like cell line, we assumed that HD11 cells may also be susceptible. To determine the replication of ALV-J in HD11 cells, we employed the qRT-PCR to quantitatively determine viral genomic RNA. According to the growth curves determined by qRT-PCR, ALV-J began to replicate at 6 hpi, indicating an eclipse period of 6 h in HD11 cells (**Figure 1A**). To further substantiate the ALV-J replication in HD11 cells, TCID_50_ assay was performed using the specific anti-gp85 antibody. As a result, the TCID_50_ curve also demonstrated a consistently increasing trend line of ALV-J, yielding a trend line similar to that determined by qRT-PCR (**Figure 1B**). According to the growth curves, ALV-J abundance reached a maximum at 36 hpi in HD11 cells. All these data indicated that ALV-J can replicate in HD11 cells.

### ALV-J Infection Attenuates the Type I Interferon Production

**FIGURE 2:**
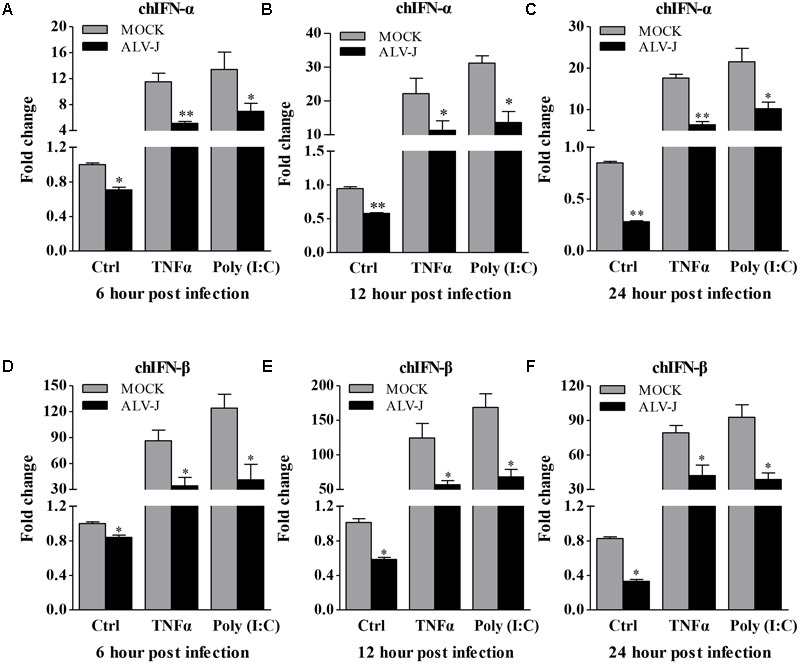
ALV-J inhibit the type I interferon production in HD11 cells. HD11 cells were infected with ALV-J or medium as a control, and then treated with TNF-α or Poly (I:C) or with DMEM medium as a control. At 6, 12, and 24 hpi, total RNA were isolated and subjected to qRT-PCR to detect the transcription profile. Expression levels of chIFNα **(A–C)** and chIFNβ **(D–F)** were calculated relative to the expression of the GAPDH gene and expressed as fold increase or decrease relative to the control samples. Results are representative of three independent experiments. Data are represented as means ± SD. ^∗∗^*P* < 0.01; ^∗^*P* < 0.05.

Because ALV-J is considered as an immunosuppressive agent (Feng and Zhang, 2016) and because type I interferon plays an important role in the host immune response against ALV-J infection (Dai et al., 2016), it was intriguing to examine the effect of ALV-J on type I interferon expression. So we detected the production of type I interferon in HD11 cells with or without ALV-J infection using qRT-PCR assay. Interestingly, we found that the transcriptional levels of IFN-α and IFN-β were significantly decreased in ALV-J-infected cells compared to that of controls either in the presence or in the absence of TNF-α or Poly (I:C) (*p* < 0.05) at 6, 12 and 24 hpi (**Figures 2A–F**), indicating the inhibitory effect of ALV-J infection on type I interferon production in HD11 cells.

**FIGURE 3:**
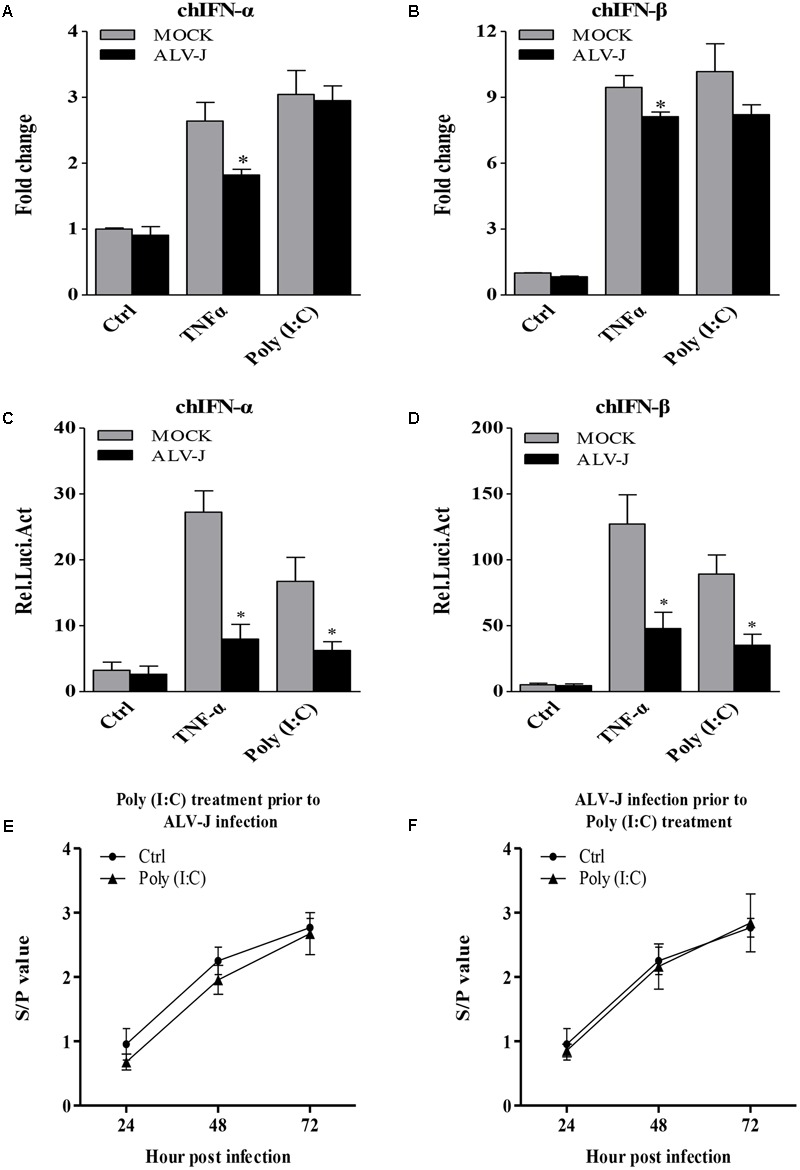
ALV-J inhibit the type I interferon production in DF-1 cells. **(A,B)** DF-1 cells grown into a monolayer were infected with ALV-J or medium as a control. Twenty-four hours later, cells were treated with TNF-α or Poly (I:C) or with medium as a control. Twelve hours after TNF-α or Poly (I:C) treatment, total RNA were isolated and subjected to qRT-PCR to detect the transcription profile. Expression levels of chIFNα and chIFNβ were calculated relative to the expression of the GAPDH gene and expressed as fold increase or decrease relative to the control samples. **(C,D)** Mock- or ALV-J-infected DF-1 cells were co-transfected with pGL3-chIFNα1 or pGL3-chIFNβ and pRL-TK Renilla reporter plasmid. Cells were treated with TNF-α or Poly (I:C) or with DMEM medium as a control at 24 hpi. Twelve hours after TNF-α or Poly (I:C) treatment, luciferase activities were detected using the dual-luciferase assay system (Promega). **(E,F)** DF-1 cells were treated with Poly(I:C) prior to ALV-J infection or infected with ALV-J prior to Poly(I:C) transfection. At 24, 48, and 72 hpi, cell cultures were collected to determine viral loads using AC-ELISA in anti-p27 antibody-coated plates (IDEXX). Results are representative of three independent experiments. Data are represented as means ± SD. ^∗^*P* < 0.05.

To determine whether the inhibitory effect also occurred in DF-1 cells, we detected the transcriptional levels of IFN-α and IFN-β. Surprisingly, ALV-J also inhibited type I interferon production in DF-1 cells in the presence of TNF-α (**Figures 3A,B**), indicating the inhibitory effect is not cell-type specific. To consolidate these findings, we employed luciferase reporter gene assay to examine the effect of ALV-J on the promoter activities of IFN-α and IFN-β. As a result, ALV-J significantly inhibited the promoter activities of IFN-α and IFN-β following stimulation with TNF-α or Poly (I:C) (**Figures 3C,D**). All these data indicated that ALV-J also possesses the ability to attenuate type I interferon production in DF-1 cells.

Considering the inhibitory effect of ALV-J on Poly (I:C)-induced type I interferon production, we expanded our investigation to study whether Poly (I:C) could inhibit ALV-J replication. As a result, ALV-J can replicate in the presence of poly(I:C) as efficiently as in the absence of poly(I:C), although poly(I:C) efficiently induces interferon (**Figures 3E,F**). These findings indicated that the inhibition of type I interferon expression by ALV-J allows ALV-J to replicate efficiently regardless of the Poly(I:C) treatment.

### mRNA Expression Profiles

**FIGURE 4:**
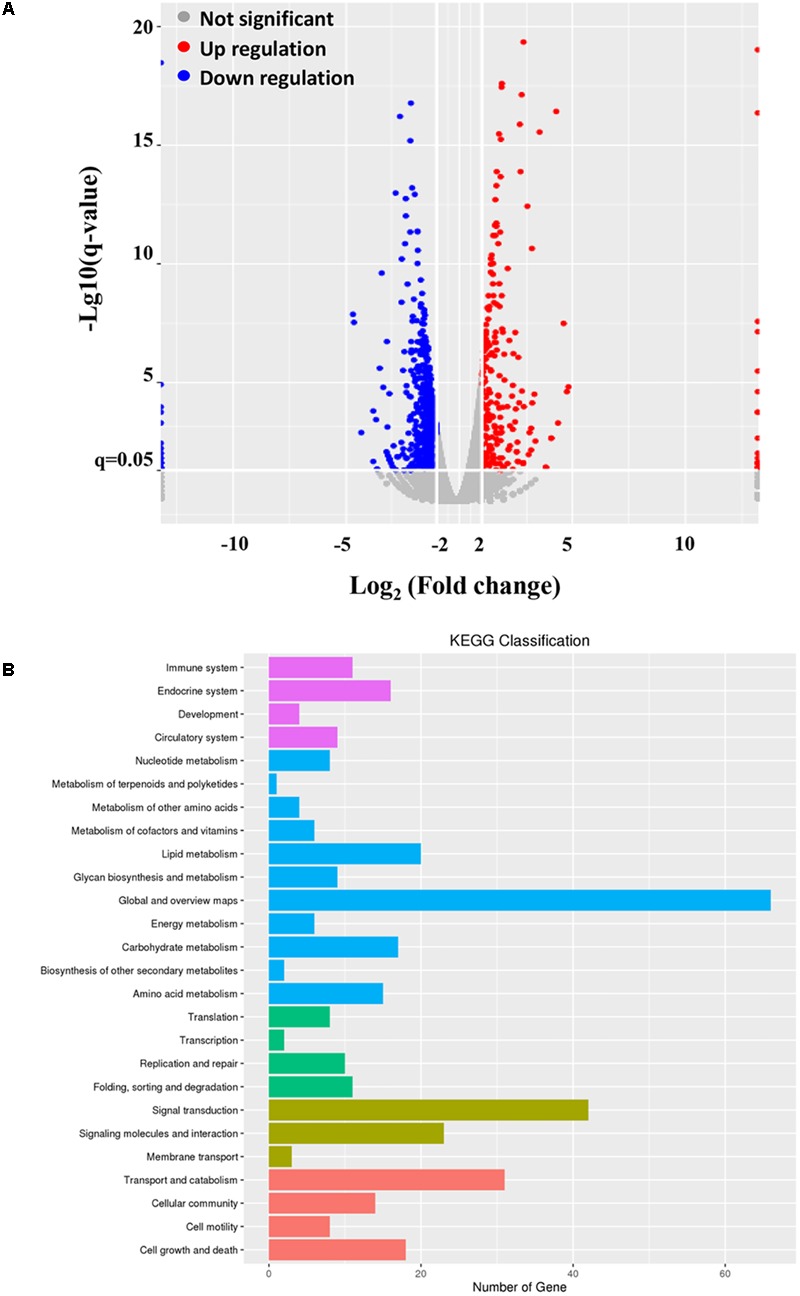
mRNA-Seq data. HD11 cells were infected with ALV-J or medium as a control. Twelve hours later, total RNA were isolated from the mock- and ALV-J-infected cells for mRNA sequencing on an Illumina Hiseq 2500 platform. **(A)** Distribution of the differentially expressed genes in ALV-J-infected HD11 cells compared to that in mock-infected HD11 cells. **(B)** KEGG classification analysis in ALV-J-infected HD11 cells compared to that in mock-infected HD11 cells.

To reveal the underlying mechanism of the inhibitory effect of ALV-J on type I interferon production, we introduced the mRNA-Seq assay in the present study. As a result, 77343682 reads and 94173664 reads were obtained from the ALV-J-infected and mock-infected HD11 cells, respectively. Genome mapping analysis showed that 72737976 mapped reads were identified in ALV-J-infected HD11 cells with mapped ratios of 90.72%, while 89446324 mapped reads were identified in mock-infected cells with mapped ratios of 91.17%. The significantly differentially expressed genes were identified by screening the DAVID database. Compared to the transcriptional levels in the mock-infected cells, 909 significantly differentially expressed genes were identified in the ALV-J-infected cells (threshold: corrected *P* < 0.05 and ratio ≥1), including 228 up-regulated genes and 681 down-regulated genes (**Figure 4A**).

To deeper understand the biological process of ALV-J infection in HD11 cells, functional analysis of the significantly differentially expressed genes was performed based on the Gene Ontology (GO) project using the SBC Analysis System. Interestingly, the differentially expressed genes were mainly involved in the following biological process: cell growth and death, immune system, transport and catabolism, signal transduction, replication and repair (**Figure 4B**). Among these differentially expressed genes, 4 differentially expressed genes (TNFSF10, IκBα, PIDD1 and RNASEL) were selected for a further study. Biological pathway and gene function analysis indicated that IκBα and RNASEL are involved in innate immunity response, while PIDD1 and TNFSF10 are involved in apoptosis (**Table 2**).

**Table 2:** Biological functions of selected genes of interest in the present study.

Gene symbol	Gene description	Function	Fold change
IκBα	NF-kappa-B inhibitor alpha	Protein hetero-/homo-dimerization activity, IκB kinase activity, scaffold protein binding	1.723
TNFSF10	Tumor necrosis factor ligand superfamily member 10	Cytokine activity, tumor necrosis factor receptor binding	0.198
PIDD1	p53-induced death domain-containing protein 1	Death receptor binding	0.283
RNASEL	2-5A-dependent ribonuclease	RNA binding, protein kinase activity, endoribonuclease activity	0.384

### Validation of the Differentially Expressed Genes

**FIGURE 5:**
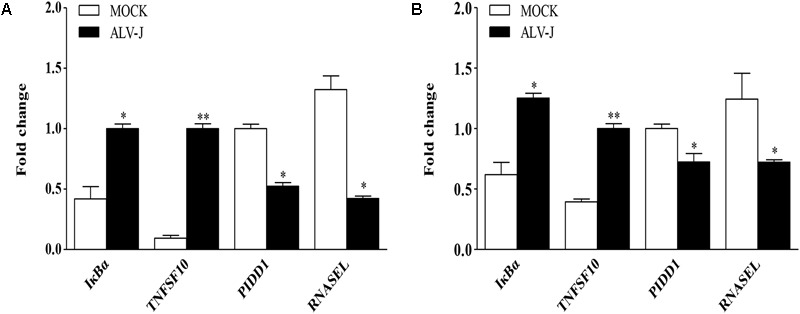
Validation of differentially expressed genes identified by mRNA-Seq. HD11 cells **(A)** or DF-1 cells **(B)** were infected with ALV-J or medium as a control. Total RNA was isolated and subjected to qRT-PCR to validate the differentially expressed genes identified by mRNA-Seq. Expression levels of target genes were calculated relative to the expression of the GAPDH gene and expressed as fold increase or decrease relative to the control samples. Results are representative of three independent experiments. Data are represented as means ± SD. ^∗∗^*P* < 0.01; ^∗^*P* < 0.05.

Although mRNA-Seq possesses the ability to measure transcription levels, which is usually used to characterize gene functions associated with transcript’s abundance, the validity of mRNA-Seq analysis still need to be confirmed using diverse ways. Therefore, we employed qRT-PCR to determine the differentially expressed genes. As a result, the transcriptional levels of IκBα and TNFSF10 were significantly up-regulated in ALV-J-infected HD11 cells compared to that in the mock-infected cells, while PIDD1 and RNASEL were significantly down-regulated in ALV-J-infected HD11 cells, yielding change trends similar to that determined by mRNA-Seq (**Figure 5A**). Similar results were obtained in an independent experiment using DF-1 cells (**Figure 5B**), indicating that the differential expression is not cell type-specific. The variation tendency of target genes showed consistent trends with the results of mRNA-Seq analysis, indicating a good indicators of mRNA-Seq analysis in the whole variations of the gene expression.

### ALV-J Causes the Accumulation of IκBα and Inhibits the Expression of NF-κB

**FIGURE 6:**
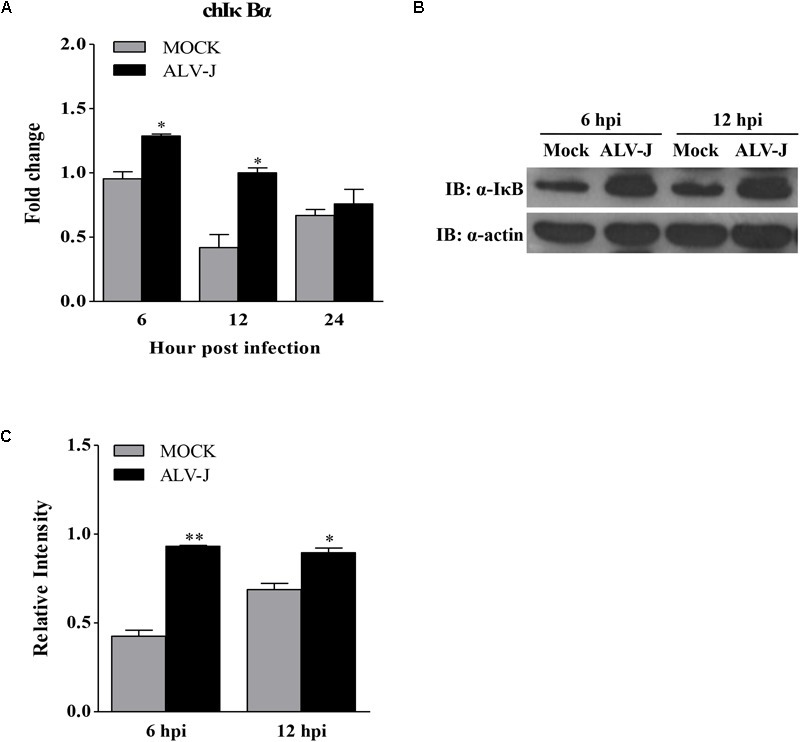
The effect of ALV-J on the expression of IκBα. **(A)** HD11 cells were infected with ALV-J or medium as a control. At 6, 12, and 24 hpi, total RNA were isolated and subjected to qRT-PCR to detect the transcription profile. Expression levels of chicken IκBα was calculated relative to the expression of the GAPDH gene and expressed as fold increase or decrease relative to the control samples. The graphs show the averages of fold changes from three individual experiments. Results are representative of three independent experiments. Data are represented as means ± SD. ^∗^*P* < 0.05. **(B)** HD11 cells infected with ALV-J or medium as a control were harvested at 6 and 12 hpi and examined by Western Blot using anti-IκBα and anti-actin antibodies. **(C)** Relative levels of IκBα in HD11 cells. The relative levels of IκBα were calculated as follows: density of bands of IκBα / band density of β-actin. Data are represented as means ± SD. ^∗∗^*P* < 0.01; ^∗^*P* < 0.05.

To determine the expression level of IκBα in ALV-J-infected HD11 cells, we detected the expression of IκBα using qRT-PCR and Western Blot. As a result, ALV-J significantly enhanced the transcriptional level of IκBα at 6 and 12 hpi, but caused little change at 24 hpi (**Figure 6A**). Similar results were obtained in an experiment using Western Blot (**Figures 6B,C**).

**FIGURE 7:**
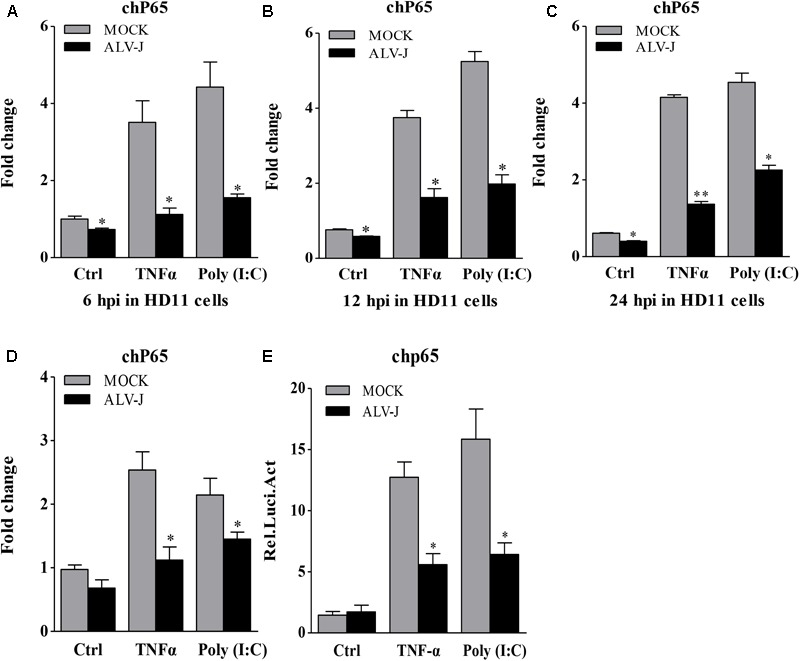
The effect of ALV-J on the expression of NF-κB p65. **(A–C)** HD11 cells were infected with ALV-J or medium as a control, and then treated with TNF-α or Poly (I:C) or with medium as a control. At 6, 12, and 24 hpi, total RNA were isolated and subjected to qRT-PCR to detect the transcription profile. Expression levels of NF-κB p65 were calculated relative to the expression of the GAPDH gene and expressed as fold increase or decrease relative to the control samples. **(D)** DF-1 cells grown into a monolayer were infected with ALV-J or medium as a control. Twenty-four hours later, cells were treated with TNF-α or Poly (I:C) or with medium as a control. Twelve hours after TNF-α or Poly (I:C) treatment, total RNA were isolated and subjected to qRT-PCR to detect the transcription profile. Expression levels of NF-κB p65 were calculated relative to the expression of the GAPDH gene and expressed as fold increase or decrease relative to the control samples. **(E)** DF-1 cells were co-transfected with pGL3-chp65 and pRL-TK Renilla reporter plasmid, followed by infection of medium or ALV-J with the indicated dose. Cells were treated with TNF-α or Poly (I:C) or with DMEM medium as a control after transfection. Twelve hours after TNF-α or Poly (I:C) treatment, luciferase activities were detected using the dual-luciferase assay system (Promega). Results are representative of three independent experiments. Data are represented as means ± SD. ^∗∗^*P* < 0.01; ^∗^*P* < 0.05.

Because ALV-J inhibited the expression of type I interferon and because IκBα accumulated during ALV-J infection plays a critical role in mediating activation of NF-κB and because type I interferon are usually regulated by NF-κB signaling pathway (Nomura et al., 2000; Gil et al., 2004), ALV-J was assumed to affect the expression of NF-κB. To test this hypothesis, we examined the transcriptional levels of NF-κB p65 in cells with or without ALV-J infection using qRT-PCR. As a result, ALV-J effectively lowered the transcriptional level of NF-κB p65 at 6, 12, and 24 hpi in HD11 cells either in the presence or in the absence of TNF-α or Poly (I:C) (**Figures 7A–C**). Similar results were obtained in an experiment using DF-1 cells (**Figure 7D**). To consolidate these findings, we examined the effect of ALV-J on the promoter activities of NF-κB p65 using luciferase reporter assay. As shown in **Figure 7E**, ALV-J significantly inhibited the promoter activities of NF-κB p65 following stimulation with Poly (I:C) or TNF-α, indicating the inhibitory effect of ALV-J on the transcriptional activity of NF-κB p65. All these data indicated that ALV-J causes the accumulation of IκB and inhibits the expression of NF-κB, which might lead to the inhibitory effect on type I interferon production.

### ALV-J Inhibits Phosphorylation of IκBα on Ser32/36 in HD11 Cells

**FIGURE 8:**
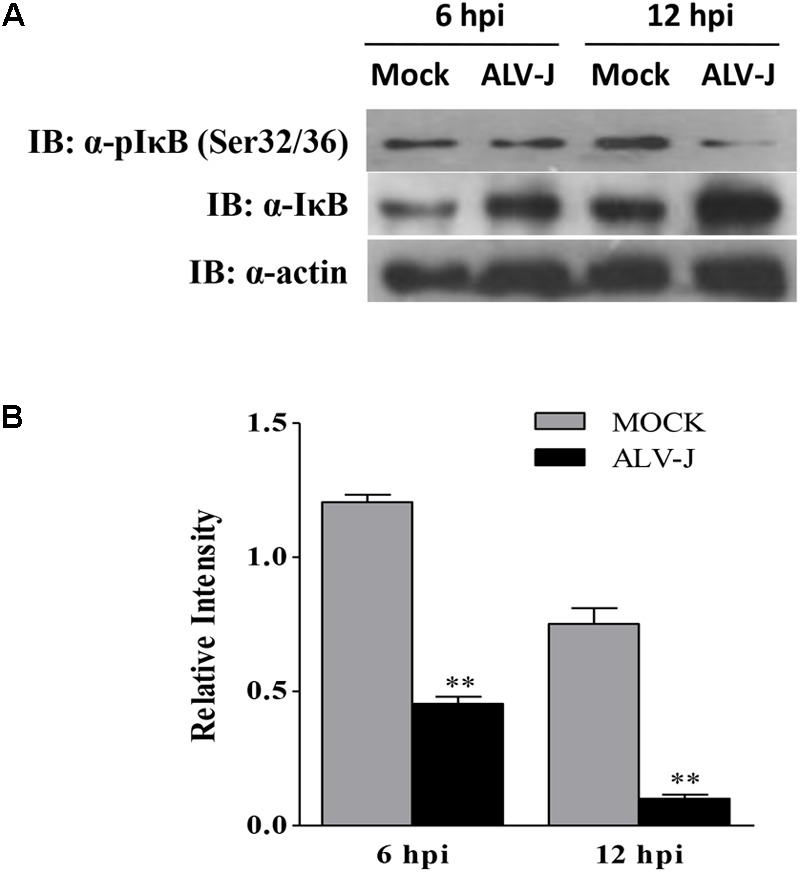
ALV-J blocked the phosphorylation of IκBα on Ser32/32. **(A)** HD11 cells were infected with ALV-J or medium as a control. Cell lysates were prepared at 6 and 12 hpi, and examined by Western Blot using anti-pIκBα (Ser32/36), anti-IκBα and anti-actin antibodies. **(B)** Relative levels of IκBα phosphorylation in HD11 cells. The relative levels phosphorylated IκBα were calculated as follows: (density of bands of phosphorylated IκBα/band density of β-actin)/(density of bands of IκBα/band density of β-actin). Data are represented as means ± SD. ^∗∗^*P* < 0.01.

IκB is usually phosphorylated by the activated IKK in the early response to pathogenic infection, leading to the dissociation of the interaction between NF-κB and IκB. Then phosphorylated IκB is degraded through ubiquitination- and proteasome-dependent degradation pathway (Baldwin, 1996; Nomura et al., 2000; Smith et al., 2014). Considering the accumulation and the degradation pathway of IκBα, we expanded our investigation to study whether ALV-J affect the IκBα phosphorylation. As a result, ALV-J caused significant decrease in the IκBα phosphorylation on Ser 32/36 amino acid residues compared to that in mock-infected cells (**Figure 8**). Thus, we propose that, when host cells are infected with ALV-J, the IκBα phosphorylation is blocked, leading to the IκBα accumulates in the cytoplasm.

## Discussion

ALV-J is an oncogenic retrovirus associated with tumorigenesis and immunosuppression. Chickens infected with ALV-J usually suffer from severe immunosuppression, leading to an enhanced susceptibility to other avian diseases. Although ALV-J has been studied intensively and considered as an immunosuppressive agent, our understanding of the mechanisms of ALV-J-induced immunosuppression is still limited.

Up to now, it has been reported that ALV-J can proliferate in multiple chicken cells, such as DF-1 cells, primary chicken embryo fibroblast (CEF), natural killer (NK) cells, DCs and macrophages (Landman et al., 2002; Liu et al., 2016b; Feng M. et al., 2017; Feng W. et al., 2017). Although DF-1 cells and CEF are commonly used for the replication of ALV-J, no overt changes (tumor growth or cytopathic effect) were observed in these cells upon ALV-J infection, so both cells are difficult to be used as the target cell model for the research of pathogenic mechanisms (Feng W. et al., 2017). As for NK cells, only one paper reported that NK cells were susceptible to ALV-J, with reduced killing activity (Landman et al., 2002). DCs are professional antigen-presenting cells in the initiation of the immunity, possessing the unique capacity to initiate primary immune responses (Geissmann et al., 2010). It has been reported that ALV-J inhibits the differentiation and maturation of chicken DCs, triggers apoptosis and alters cytokine expression (Wu et al., 2010; Liu et al., 2016a,b). Recently, a report indicated that chicken primary monocyte-derived macrophages (MDM) were susceptible to ALV-J, with the differential expression of antiviral genes and inflammatory genes (Feng M. et al., 2017). but this study did not provide any solid evidence for the immunosuppression caused by ALV-J. However, these results provide clues that macrophage might be used as a cell model to research the ALV-J-induced immunosuppression. HD11 cells, developed by transformation of the chicken bone marrow cells with a replication-defective retrovirus, possess normal macrophage functions and markers including phagocytosis, ATPase activity, macrophage surface antigens, Fc receptors and cytokine secretion ability (Beug et al., 1979; Lyon and Hinshaw, 1991). Therefore, HD11 cell have been widely used for the research of avian pathogens, such as influenza A virus, IBDV, ALV and *Salmonella* (Beug et al., 1979; Lyon and Hinshaw, 1991; Lam, 1998; Liniger et al., 2012; Park et al., 2013). Considering the critical role of macrophages in the process of the retrovirus infection (Campbell et al., 2014) and the macrophage characteristics of HD11 cell, we employed the HD11 cell as the target cell model to examine the effects of ALV-J on the innate immune response in the present study. In this study, we determined the growth curves of ALV-J with qRT-PCR and TCID_50_ assay. The TCID_50_ curve describes a consistently increasing trend line of ALV-J infectious virus, yielding a trend line similar to that determined by qRT-PCR, indicating the susceptibility of HD11 cells to ALV-J.

ALV-J is considered as an immunosuppressive agent (Feng and Zhang, 2016). It has been reported that the expression of type I interferon (IFN-α and IFN-β) and some pro-inflammatory genes (IFN-γ, IL-6, IL-10, and IL-1β) show no significant changes from 1 to 7 dpi *in vivo* (Gao et al., 2015; Feng et al., 2016). There are several lines of evidence showing that ALV-J can induce the innate response. However, we first found that ALV-J inhibits type I interferon production in host cells in the present study. It seems that the effect of ALV-J on type I interferon production *in vivo* and *in vitro* is contradictory. Actually, the conflicting results might be due to the infection model or the infection process. After all, ALV-J-induced immunosuppression may involve multiple factors, and these findings *in vivo* were observed in a specific infection stage or due to a lack of systematic analysis. More effort will be required to systematically reveal the effect of ALV-J on the innate response.

High-throughput mRNA sequencing assay possess the ability to measure transcript expression levels in a single assay, which is usually used to characterize gene functions associated with transcript’s abundance (Mortazavi et al., 2008; Nagalakshmi et al., 2008). Host gene expression profiles following ALV-J infection have been screened using this assay in chickens, DF-1 cells and DCs (Yang et al., 2007; Fan et al., 2012; Liu et al., 2016a; Zhang et al., 2016). To reveal the underlying mechanism of the inhibitory effect of ALV-J, mRNA-Seq assay was introduced in this study. According to the mRNA-Seq analysis, a total of 909 significantly differentially expressed genes were identified, including 228 up-regulated genes and 681 down-regulated genes. Among these differentially expressed genes, we focused on a vital gene IκBα because of the critical role of IκB in mediating activation of NF-κB (Nomura et al., 2000; Gil et al., 2004). The finding provide clues that ALV-J-induced immunosuppression might be associated with NF-κB signaling pathway.

Till now, there are still no reports about the effect of ALV-J on NF-κB signaling pathway. NF-κB serves as a molecular sensor responding to viral infection such as that with human immunodeficiency virus (Demarchi et al., 1999), Theiler’s murine encephalomyelitis virus (Palma et al., 2003) and measles virus (Helin et al., 2002). In contrary, some viruses(FMDV and EMCV) inhibit NF-κB signaling pathway to antagonize the host response (Li X. et al., 2013). Considering the regulated function of NF-κB signaling pathway on type I IFN expression, the effect of ALV-J on NF-κB signaling pathway was determined. Interestingly, we found that ALV-J effectively lower the expression level of NF-κB and significantly enhance the expression level of IκBα at different time points in host cells. It has been reported that IκB sequestering NF-κB in the cytoplasm is considered as a critical brake on NF-κB signaling, IκB can be phosphorylated by IKKs in response to the extracellular stimuli, leading to its degradation and subsequent NF-κB activation (Baldwin, 1996; Napetschnig and Wu, 2013). In the present study, the observation that ALV-J cause the increased expression of IκBα give us a clue that IκB phosphorylation might be blocked in the HD11 cells infected with ALV-J. Our further study also confirmed this assumption, as ALV-J blocked the phosphorylation of IκBα on Ser 32/36 amino acid residues, leading to the stabilization of NF-κB/ IκB complex in the cytoplasm. Our findings clearly show that the inhibitory effect of ALV-J infection on type I interferon is associated with transcriptional regulator NF-κB. Accordingly, when host cells were infected with ALV-J, the IκBα phosphorylation is blocked, causing the IκBα accumulation in the cytoplasm. The accumulated IκBα make NF-κB/IκB complex stabilized, prevent the NF-κB transferring into the nucleus, and finally suppress the interferon expression.

Of note, the mechanism underlying the immunosuppression induced by pathogenic infection may vary (Li Z. et al., 2013). Here, we reported the effect of ALV-J on the type I interferon production and the activation of NF-κB signaling pathway. The suppression of cytokine expression might be only one of the tricks exploited by ALV-J to evade innate immune response. Therefore, several questions are raised. For example, how does ALV-J inhibit the phosphorylation of IκB? Which component (viral protein, nucleotide or something else) of ALV-J affect type I interferon expression and the activation of the NF-κB? Viruses always antagonize host innate immune response via interaction with a critical protein in the signaling pathway. The genome of ALV-J encodes more than ten proteins, but which viral protein are interacting with IκB phosphorylation is unclear. Therefore, we propose that viral proteins of ALV-J might interact with IκB, cover its phosphorylated site and block the phosphorylation of IκB, causing its accumulation in the cytoplasm. Of course, More effort will be required to answer these questions.

In summary, our results reveals that ALV-J possesses the inhibitory effect on type I interferon production in HD11 cells, which might be associated with the transcriptional regulator NF-κB. The findings that ALV-J significantly blocked the phosphorylation of IκB and inhibited the expression of NF-κB suggest that IκB serves as a negative regulator in ALV-J-induced immunosuppression. Mechanistically, ALV-J attenuated type I interferon production by blocking the phosphorylation of IκB, which stabilized the NF-κB/IκB complex and prevented the transfer of NF-κB into the nucleus. These findings provided insights for further studies of the molecular mechanism of ALV-J infection.

## Author Contributions

WL, HZ, and QX designed the research. WL, ZX, YY, HL, and WC performed and analyzed the experiments. WL, ZX, and FC analyzed the data. WL and QX wrote the manuscript draft. All authors reviewed the manuscript.

## Conflict of Interest Statement

The authors declare that the research was conducted in the absence of any commercial or financial relationships that could be construed as a potential conflict of interest.
